# STA–MCA Bypass for Moyamoya Vasculopathy: Early Experience and Outcomes from a National Referral Centre

**DOI:** 10.21315/mjms-05-2026-294

**Published:** 2026-08-31

**Authors:** Sarah Atiqah Mohd Zamri, Nurfaten Hamzah, Noor Ain Najwa Mohd Firdaus, Thinesh Kumaran Jayaraman

**Affiliations:** 1 Department of Neurosurgery, Tunku Abdul Rahman Neuroscience Institute, Hospital Kuala Lumpur, Jalan Pahang, 50586 Kuala Lumpur, Malaysia; 2 Department of Neurosciences, School of Medical Sciences, Health Campus, Universiti Sains Malaysia, 16150 Kubang Kerian, Kelantan, Malaysia

**Keywords:** cerebrovascular surgery, revascularisation surgery, cerebral bypass surgery, moyamoya disease

## Abstract

**Background:**

Moyamoya vasculopathy is a progressive cerebrovascular disorder requiring surgical revascularisation. In Malaysia, experience with cerebral bypass has been limited. This study evaluated early outcomes of superficial temporal artery–middle cerebral artery (STA–MCA) bypass at a national referral centre.

**Methods:**

A retrospective study was conducted on patients undergoing STA–MCA bypass between January 2017 and December 2025. Functional outcomes were assessed using the modified Rankin Scale (mRS), while cerebral perfusion and bypass patency were evaluated using computed tomography (CT) perfusion and CT angiography or digital subtraction angiography. Statistical analyses included Fisher’s exact test, Mann–Whitney U test, and Spearman correlation.

**Results:**

A total of 74 cerebral bypass procedures were performed, including 50 for moyamoya vasculopathy, with 37 cases analysed. Median age was 43 years with female predominance (54%). Single-barrel bypass was performed in 84% of cases, with 100% final intraoperative patency. Perfusion improved in 85% of patients and graft patency in 94%. At three months, 95% achieved good functional outcome (mRS 0 to 2). Greater surgeon experience was associated with shorter operative duration (ρ = −0.463, *P* = 0.0039). Preoperative antiplatelet withholding was also associated with reduced operative duration (*P* = 0.0109) without adverse impact on clinical or radiological outcomes. No variables were significantly associated with adverse outcomes (*P* > 0.05).

**Conclusion:**

STA–MCA bypass achieves excellent clinical and radiological outcomes in a maturing neurovascular programme. These findings support the development of structured revascularisation services and inform perioperative management strategies in a national referral setting.

## Introduction

Moyamoya disease is a chronic, progressive cerebrovascular steno-occlusive disorder involving the terminal internal carotid arteries and their proximal branches (1). Patients commonly present with ischaemic stroke or transient ischaemic attacks, and intracranial haemorrhage (1, 2). The disease is characterised by the development of abnormal basal collateral networks (moyamoya vessels), which are hemodynamically insufficient and structurally fragile, conferring risk of hypoperfusion and haemorrhage from collateral rupture (1, 3). Although initially described predominantly in East Asian populations, moyamoya is increasingly recognised outside traditional high-incidence regions, including Southeast Asia, likely reflecting growing clinical awareness and wider availability of advanced neurovascular imaging; in Malaysia, cases have been documented for decades (4–6).

Surgical revascularisation, most notably superficial temporal artery–middle cerebral artery (STA–MCA) bypass, has become the cornerstone of definitive treatment for moyamoya disease. Direct bypass aims to immediately augment cerebral blood flow and reduce hemodynamic stress on fragile collateral networks. In haemorrhagic moyamoya, the Japan Adult Moyamoya Trial demonstrated a 60% reduction in rebleeding rates following extracranial–intracranial bypass, and long-term outcome studies have reported 5-year stroke-free survival rates of 90% to 95% in patients undergoing bypass than those managed conservatively (7). Long-term follow-up studies of STA–MCA bypass have reported very high rates of freedom from stroke or death over extended observation periods, reinforcing the durability of direct revascularisation (8). Meta-analyses further confirm that direct bypass or combined procedures are associated with lower recurrent stroke risk and superior angiographic outcomes compared with indirect bypass alone, with direct techniques yielding better prevention of future cerebrovascular events (9).

Historically, Malaysia lacked a centralised protocol or experience base for moyamoya surgery, leading to fragmented care and underutilisation of revascularisation. At Hospital Kuala Lumpur, this paradigm has shifted significantly over the past decade. With the leadership of a dedicated neurovascular team and strategic knowledge transfer from international mentors, the department has evolved from a cautious adopter to a high-volume centre of cerebral bypass surgery. This progress reflects not only growing surgical expertise, but also the integration of advanced neuroimaging, structured perioperative care, and multidisciplinary collaboration.

This study presents a comprehensive retrospective analysis of our institutional experience in STA–MCA bypass surgery for moyamoya disease. To our knowledge, at current moment, this is the first and largest reported series of adult moyamoya bypass surgeries in Malaysia, reflecting the experience of the national referral centre with the country’s highest neurovascular case volume. We reviewed patient demographics, surgical laterality, radiological outcomes, functional recovery, and complication profiles. Through this analysis, we aimed to identify key trends, validate treatment efficacy, and formalise a departmental protocol that could serve as both a clinical guide and a training framework anchored in data, strengthened by outcomes, and aligned with global standards of care.

## Methods

This is a retrospective, single-centre observational study conducted in the Neurosurgery Department at Hospital Kuala Lumpur, Malaysia, involving patients diagnosed with moyamoya vasculopathy who underwent superficial temporal artery to middle cerebral artery (STA–MCA) bypass surgery. Total yearly data of neurovascular patients who received microsurgical or endovascular intervention were identified. These cases were narrowed down to cerebral bypass in general and, specifically, bypass for moyamoya. Only direct bypass cases were included in this study.

### Data Collection and Analysis

Patient data were retrieved from hospital medical records and operative logs. Variables collected included demographic characteristics, presenting symptoms, laterality and type of bypass, perioperative characteristics, intraoperative findings, and postoperative outcomes. Staging of the disease was based on radiological grading known as Suzuki Grading and computed tomography (CT) perfusion staging. Patients chosen for surgery were all symptomatic, presenting with ischaemic or haemorrhagic stroke, and recurrent transient ischaemic attack. Functional outcomes were assessed using the modified Rankin Scale (mRS) while cerebral perfusion status was evaluated using pre- and postoperative CT perfusion imaging when available. Bypass patency was assessed through postoperative CTA or digital subtraction angiography (DSA) imaging.

Data were analysed using IBM SPSS Statistics (version 27; IBM Corporation). Variables were presented as frequencies and percentages. Univariate analyses were performed to evaluate associations between clinical variables and each outcome separately. Fisher’s exact test was used for categorical data, and the Mann–Whitney U test was used for continuous variables. Univariate associations across all outcomes were summarised in a single table reporting *P*-values only. All statistical tests were two-sided, and *P* < 0.05 was considered statistically significant. Given the exploratory nature of the study and the limited sample size, results were interpreted with caution.

### Pre-operative Preparation

All patients underwent standard preoperative workup, including MRI, DSA, CT angiography and CT perfusion imaging to assess cerebral hemodynamics. Antiplatelet therapy, initiated preoperatively, was withheld in selected cases only; others on single antiplatelet therapy were continued, and hydration protocols were optimised to minimise perioperative ischaemic risk.

### Surgical Technique

All surgeries were performed under general anaesthesia. A frontotemporal straight vertical incision was made to harvest the parietal or/and frontal branch of the STA. The recipient cortical branch of the MCA was identified via a small temporal bone craniotomy. Donor and recipient were prepared. An end-to-side anastomosis was performed using interrupted 9-0 or 10-0 nylon sutures. Intraoperative fluorescein sodium angiography and vascular Doppler were used to confirm flow across the anastomosis (Figure 1). The bone flap was replaced without compression of the bypass.

### Postoperative Management

Patients were managed in the neurocritical care unit for hemodynamic monitoring and blood pressure control. Antiplatelet therapy was resumed within 24 h postoperatively if it was withheld. Postoperative imaging CT brain was performed on day one after surgery and followed with angiographic as well as perfusion studies at six months. Neurological status and mRS scoring were assessed clinically at follow-up visits.

Following the comprehensive analysis of surgical outcomes, radiological changes, and perioperative challenges, a formal departmental protocol for the management of moyamoya disease was developed to guide future clinical practice and training.

## Results

The Department of Neurosurgery at Hospital Kuala Lumpur, Malaysia, is one of the largest national referral centres for neurovascular disease, particularly for complex cases. Since 2017, the department has managed an average of approximately 150 to 200 neurovascular cases annually through microsurgical and/or endovascular treatment. Between 2022 and 2025, a total of 770 neurovascular cases underwent microsurgical or endovascular management at our institution, with annual caseloads of 176, 221, 175 and 198 cases, respectively.

Since the initiation of bypass surgery at our institution in 2017, a total of 74 cerebral bypass procedures were performed through December 2025, with annual volumes increasing from four cases in 2017 to 13 cases in 2025. Of these, 50 procedures were undertaken for moyamoya disease. For the purposes of statistical analysis, only cases performed between 2017 and 2024 were included. Patients who underwent bypass surgery in 2025 were excluded due to incomplete clinical and/or radiological follow-up at the time of analysis. Consequently, the final study cohort comprised 37 moyamoya bypass cases with complete follow-up data available for outcome assessment. Tables 1 and 2, and Figure 2 provide a comprehensive overview of cerebral revascularisation surgeries performed for moyamoya disease at Hospital Kuala Lumpur between 2017 and 2025.

As illustrated in Figure 2, the volume of bypass surgery for moyamoya disease showed a sustained contribution to the overall institutional bypass workload during the study period.

The cohort (2017–2024) comprised 37 patients with moyamoya disease, with a slight female predominance (54%) and a median age of 43.0 years (IQR 32.0 to 51.0). Most patients were between 30 and 60 years of age (81%). The cohort was ethnically diverse, reflecting the national population distribution, with Malay (49%), Chinese (38%), and Indian (13%) patients represented. Comorbid conditions were common, present in 78% of patients, most frequently hypertension, dyslipidaemia, and diabetes mellitus. Surgical intervention was performed slightly more often on the left hemisphere (56.8%) than the right. Single-barrel bypass constituted the predominant surgical strategy (84%) while double-barrel bypass was reserved for selected cases (16%).

Most procedures were performed by two high-volume neurosurgeons, reflecting a centralised surgical practice model. This minimises inter-operator variability and ensures reduced bias in outcome assessment. Antiplatelet therapy was withheld preoperatively in most patients (86.5%). Operative duration was consistent across cases, with a median duration of 4.0 h (IQR 4.0 to 4.0), ranging from 2 to 5 h.

Immediate intraoperative bypass patency was achieved in 78.4% of cases while the remaining 21.6% required adjunct manoeuvres to establish flow. Following these manoeuvres, final intraoperative patency was achieved in all cases (100%).

Postoperative clinical events occurred in 21% of patients, most commonly seizures and transient ischaemic attacks, with no cases of major stroke or mortality, with the majority of the events happening within the first three months post-surgery (16%) compared with the period beyond three months (two events, 5%), suggesting a temporal clustering of complications in the early postoperative phase.

Functional outcomes, assessed using the modified Rankin Scale (mRS), demonstrated progressive improvement over time. Preoperatively, 27 patients (73.0%) had good functional status (mRS 0 to 2) while 10 patients (27.0%) had poor functional status (mRS 3 to 6). At one month postoperative, the proportion of patients with good functional outcomes increased to 32 (86.5%), with a corresponding reduction in poor outcomes to five patients (13.5%). By three months postoperative, 35 patients (95%) achieved good functional status, and two patients (5%) remained in the poor outcome category (Table 3). Notably, this patient initially presented with severe disability (mRS 5) and improved to mRS 3 at three months follow-up.

Radiological follow-up demonstrated improvement in cerebral perfusion in 85.3% of patients, with hyperperfusion observed in one case. Six-month angiographic follow-up showed a high bypass patency rate of 94%.

### Association Between mRS Outcome, Radiological Outcomes, and Bypass Patency with Clinical Variables

Univariate analyses were performed to explore potential associations between clinical variables with functional outcomes (mRS), CT perfusion outcomes and bypass patency (Table 4). The association was measured using Fisher’s exact test for categorical variables, while continuous variables were analysed using the Mann– Whitney U test due to a small sample size. Each outcome was analysed separately.

At three months (*n* = 37), 95% of patients achieved good functional recovery (mRS 0 to 2) while 5% had poor outcomes (mRS 3 to 6). Exploratory associations testing showed no statistically significant association between mRS outcomes status and age, comorbidities, stroke presentation type, surgical side or withholding of antiplatelet therapy (all *P* > 0.05, Fisher’s exact test). A statistically significant association was observed between mRS outcomes and duration of surgery (*P*-value = 0.006, Mann–Whitney U test). Patients with good outcomes had a shorter median surgery duration of 4 h, compared with a median of 5 h in the poor outcome group (*n* = 2). Despite reaching statistical significance, this finding remained exploratory due to the small number of poor outcomes.

CT perfusion imaging follow-up at three to six months showed perfusion improvement in 85.3% of patients, unchanged perfusion in 11.8% and hyperperfusion in 2.9%. Exploratory univariate analyses demonstrated no statistically significant associations between CT perfusion outcome categories and gender, age, comorbidities, stroke presentation type, surgical side, antiplatelet withholding, or duration of surgery (all *P* > 0.05).

At six months, 33 patients underwent imaging follow-up, of whom 94% had confirmed bypass patency on CT angiography (CTA) or DSA. No statistically significant associations were observed between bypass patency and gender (*P* = 0.227), presence of any comorbidity (*P* = 0.521), type of stroke presentation (*P* = 0.616), surgical side (*P* = 0.324), or antiplatelet withholding (*P* = 0.716). Similarly, there was no significant difference in age between patients with bypass patency (*P* = 0.500). Duration of surgery also did not differ significantly between groups (*P* = 0.273). These findings indicate that, within this cohort, no demographic or clinical factor showed a measurable association with six-month bypass patency.

Formal predictive modelling was not performed due to the very small number of poor outcomes and graft occlusion events (*n* = 2). All findings should be interpreted as exploratory and hypothesis-generating, acknowledging limited statistical power, outcome imbalance and inability to support multivariable adjustment.

### Association Between Preoperative Antiplatelet Holding with Perioperative Variables

Operative duration differed between patients with and without preoperative antiplatelet withholding (Mann–Whitney U, *P* = 0.0109), with longer operative times observed in patients who did not have antiplatelet therapy withheld. However, no significant association was observed between antiplatelet withholding and intraoperative immediate bypass patency (Fisher’s exact test, *P* = 0.564). Double-barrel bypass was associated with longer operative duration compared with single-barrel bypass (Mann–Whitney U test, *P* = 0.006) (Table 5). Multivariable adjustment was not performed due to the limited number of events.

### Association of Preoperative Antiplatelet Withholding with Outcomes (with Effect Size)

Due to small cohorts, we tabulated the risk difference/relative risk (effect size) (Table 6). Preoperative withholding of antiplatelet therapy 5 to 10 days prior to surgery was not significantly associated with intraoperative immediate patency, bypass patency at six months, or postoperative clinical events (all Fisher’s exact *P* > 0.05). Effect size analysis demonstrated minimal differences between groups, with risk differences close to zero for patency outcomes and low absolute event rates for clinical events.

Surgeon case volume within the cohort was not associated with immediate intraoperative patency, six-month bypass patency, or postoperative clinical events (all *P* > 0.05). However, higher surgeon case volume was correlated with shorter operative duration (ρ = −0.463, *P* = 0.0039) (Table 7).

Associations between surgeon experience and functional outcome were explored using non-parametric tests; however, formal statistical testing was limited by the low event rate. Due to the very low number of poor functional outcomes (mRS ≥ 3), statistical association between surgeon experience and functional outcome could not be reliably assessed.

We explored multivariable modelling on few variables, but the low event rates made logistic regression inappropriate. We therefore focused on descriptive statistics, univariate analyses, and effect sizes.

To explore potential confounding by indication, additional analyses were performed to compare baseline disease severity characteristics between single-barrel and double-barrel bypass groups (Table 8). No significant association was identified between bypass configuration and CT perfusion grade (*P* = 0.351), Suzuki stage (*P* = 0.177), or preoperative functional status (mRS; *P* = 0.620).

## Discussion

This study presents the largest reported single-centre experience of STA–MCA bypass surgery for moyamoya vasculopathy in Malaysia. It documents the maturation of cerebral revascularisation practice at a national referral centre. Our findings demonstrated high technical success, durable graft patency, meaningful perfusion improvement, and excellent functional outcomes, with low perioperative morbidity and no mortality. Importantly, these outcomes were achieved despite a heterogeneous patient population with a high prevalence of medical comorbidities. The steady increase in bypass volume over the study period reflected both increased institutional experience and the selective application of cerebral revascularisation in appropriately selected patients. Rather than indiscriminate expansion, bypass surgery was incorporated in a protocolised manner, with careful patient selection and standardised perioperative management. This approach likely contributed to the consistency of outcomes observed across the cohort.

The demographic profile of our cohort was consistent with adult moyamoya populations reported in international series, with a predominance of patients in the third to sixth decades of life and a slight female preponderance (10). Karsonovich et al. (2) reported that moyamoya disease generally has a bimodal age distribution, with incidence peaks at 5 to 9 years and 45 to 49 years, reflecting adult-onset in the third to sixth decades. The majority presented with ischaemic events while nearly one quarter presented with haemorrhagic stroke, reflecting the mixed adult phenotype commonly described outside paediatric populations. The high prevalence of comorbidities, particularly hypertension, dyslipidaemia, and diabetes mellitus, highlighted the coexistence of systemic vascular risk factors in adult moyamoya patients and underscored the importance of meticulous perioperative optimisation.

### Surgical Strategy and Intraoperative Patency

Single-barrel STA–MCA bypass constituted the predominant surgical strategy, with double-barrel bypass reserved for selected patients with more extensive perfusion deficits. Immediate intraoperative patency was achieved in most cases, and all initially non-patent bypasses were successfully salvaged using adjunct manoeuvres, such as topical papaverine, arterial massage, and removal of clot or thrombectomy (if bypass occlusion). This emphasises the importance of vigilant intraoperative assessment and technical flexibility. It also supports the concept that early non-patency should be viewed as a correctable intraoperative finding rather than a procedural failure.

Several studies indicate that the choice of bypass technique in moyamoya disease is frequently guided by baseline cerebral hemodynamic and disease severity rather than inherent procedural risk. Double-barrel bypass is typically selected for patients with severe hemispheric hypoperfusion and extensive vascular compromise, reflecting more advanced disease (11). Comparative analyses have shown no significant differences in postoperative stroke, haemorrhage, or functional outcomes between double-barrel and single-branch bypass types, suggesting that baseline disease characteristics drive postoperative event rates rather than bypass type per se (12).

The association between bypass type and postoperative clinical events (*P* < 0.001) in our study likely reflects differences in baseline disease severity and surgical indication rather than an intrinsic increase in procedural risk (Table 4). Patients selected for double-barrel bypass typically had more advanced disease or wider perfusion deficits, introducing confounding by indication.

### Troubleshooting and Salvage of Early Bypass Flow Compromise

While final bypass patency is commonly reported as a marker of technical success, the process of recognising and correcting early flow compromise is rarely discussed in detail. In our experience, approximately one-fifth of bypasses required intraoperative salvage manoeuvres before satisfactory flow could be established, providing important insights into the practical challenges of cerebral revascularisation.

A notable finding in our series was that 21.6% of bypasses required intraoperative salvage manoeuvres before satisfactory flow could be established. Early bypass flow compromise may occur despite a technically completed anastomosis and is often attributable to reversible causes rather than outright technical failure. Common mechanisms include donor or recipient vessel vasospasm resulting from vessel manipulation; temporary thrombosis at the anastomotic site; sluggish flow secondary to low-pressure gradients; narrowing caused by overly tight sutures; intimal irregularity; or subtle kinking and torsion of the donor vessel. Recognition of the underlying cause is critical as each mechanism requires a different corrective strategy.

When bypass flow appeared suboptimal, initial troubleshooting focused on optimising systemic hemodynamic parameters. Mean arterial pressure was augmented by increasing the mean arterial pressure (MAP) by approximately 10% to 20% where appropriate, assessing and correcting intravascular volume status, and avoiding excessive hypocapnia to maximise cerebral perfusion and donor-to-recipient flow. Only after potentially reversible hemodynamic factors had been addressed were vasospasm, thrombosis, or technical imperfections systematically investigated.

For suspected vasospasm, warm saline irrigation and topical papaverine application were used to promote vasodilation and improve vessel calibre. Gentle vessel massage was occasionally performed to facilitate restoration of flow and relieve focal spasm. In cases of sluggish flow or suspected temporary thrombus formation, careful assessment of the anastomosis was undertaken to determine whether the underlying cause was thrombotic, vasospastic, or technical in nature. When a small donor vessel branch had been preserved during STA harvesting, this branch could be utilised as a flushing channel for heparinised saline. Retrograde and antegrade flushing through the donor vessel helped evacuate small platelet aggregates or fresh thrombus, confirm luminal patency, and reduce thrombus propagation before reassessment of bypass flow. This manoeuvre was particularly useful when thrombosis was suspected, but complete occlusion had not yet occurred, potentially avoiding unnecessary revision of an otherwise satisfactory anastomosis. If intraluminal thrombus persisted or bypass flow remained unsatisfactory, open thrombectomy was subsequently performed. Technical problems, such as narrowing of the anastomotic opening, donor vessel kinking, malalignment, twisting, inadequate bite placement or intimal irregularity, required revision of the anastomosis or adjustment of the bypass configuration. In rare situations where satisfactory flow cannot be restored despite correction of vasospasm, thrombosis, and technical imperfections, consideration should be given to a rescue bypass using an alternative donor vessel, additional bypass, or graft reconstruction, depending on the intraoperative findings. The willingness to reassess the original strategy and proceed with alternative revascularisation options is an important principle of cerebral bypass surgery, as the ultimate goal is durable cerebral revascularisation rather than completion of a predetermined anastomosis. Following these interventions, all initially compromised bypasses in our series were successfully salvaged, resulting in a final intraoperative patency rate of 100%.[Fig f3-06mjms3304_oa]

These findings emphasise that reduced bypass flow should not be immediately regarded as a technical failure. Instead, a systematic troubleshooting approach, supported by meticulous intraoperative assessment and familiarity with salvage techniques, can successfully reverse many causes of early bypass compromise. For surgeons developing expertise in cerebral revascularisation, the ability to identify and correct reversible causes of flow insufficiency represents a critical component of the bypass learning curve. It may be as important as the anastomosis itself in achieving durable long-term patency.

### Perioperative Hemodynamic Target

Perioperative hemodynamic management is typically stratified into intraoperative and postoperative phases. Intraoperatively, MAP-based targets adjusted to baseline blood pressure are most used, with median MAP goals of 70 to 100 mmHg and systolic pressures of 110 to 155 mmHg. Postoperatively, SBP is more frequently targeted, with median goals of 100 to 150 mmHg while MAP targets are generally maintained within the 70 to 100 mmHg range (13).

### Perioperative Antiplatelet

Perioperative antiplatelet management in moyamoya revascularisation remains highly variable. In an international transdisciplinary survey by Eckert et al, 42.9% of clinicians did not routinely discontinue antiplatelet therapy preoperatively, 19.1% routinely discontinued antiplatelet therapy, and 38.1% were unsure (13). This reflecting the absence of or no strong evidence-based guidance as well as the need to balance ischaemic and haemorrhagic risks. If antiplatelet agents were withheld before surgery, neurologists and neurosurgeons were more likely to resume antiplatelet therapy within 24 to 48 h postoperatively (13).

However, in our study, the absence of statistically relevant differences in withholding antiplatelet supports the perioperative safety of temporary antiplatelet withholding in selected patients undergoing bypass surgery. This means antiplatelet withholding did not adversely affect immediate technical success of the bypass, temporary cessation of antiplatelet therapy did not compromise graft durability at follow-up, and antiplatelet withholding was not associated with an increased risk of postoperative clinical events (Table 5). However, a larger number of study samples that include continuation of antiplatelet should be conducted in future.

### Postoperative Clinical Event

Postoperative clinical events after moyamoya bypass are predominantly observed in the immediate and early postoperative period, with the majority occurring within the first three months after surgery (Table 2). Multiple series report that most neurological adverse events cluster early rather than in later follow-up, and that controlling early postoperative stroke remains critical to achieving surgical benefit (14, 15). Early post-bypass complications are relatively uncommon beyond the initial months, and many patients demonstrate good functional recovery and bypass patency by medium-term follow-up. This temporal distribution suggests that perioperative and early postoperative factors play a more prominent role in complication risk than late failure.

### Functional Outcome Over Time

From our study, functional outcomes improved progressively over time, with a marked shift toward good functional status by three months postoperative (Table 3). While over one-quarter of patients had poor functional status preoperatively, nearly all patients achieved functional independence at follow-up. This temporal pattern likely reflects both immediate hemodynamic improvements following bypass and delayed neurological recovery as cerebral perfusion stabilises. Similar delayed improvement has been described in other moyamoya series, reinforcing the importance of medium and long-term follow-up when assessing surgical efficacy.

In a large surgical series by Abhinav et al. (16), revascularized patients demonstrated improved performance status (e.g., mRS) over long-term follow-up compared with preoperative status, especially when direct STA–MCA bypass was performed, supporting a pattern of improved functional independence over time (17).

### Surgeon Experience and Operative Duration

Surgeon case volume was inversely correlated with operative duration, suggesting increased procedural efficiency with experience, whereas graft patency and clinical outcomes appear to be more strongly influenced by disease characteristics and surgical complexity than by surgeon volume alone (ρ = −0.463, *P* = 0.0039) (Table 7). The association between experience and operative duration was attenuated after adjustment for bypass complexity, indicating that operative time is influenced primarily by surgical strategy rather than surgeon volume alone. This highlights the importance of accounting for procedural complexity when interpreting efficiency metrics.

This is consistent with broader surgical literature; surgeon case volume and experience have been associated with greater procedural efficiency and reduced operative duration in a number of settings. Systematic reviews across diverse surgical disciplines demonstrate that increased individual surgeon volume correlates with improved performance metrics, including shorter operative times (18).

However, outcomes such as graft patency and clinical results are more closely linked to disease complexity and surgical strategy than volume alone. Morche J et al. (19) supported this observation, emphasising that procedure-specific volume–outcome relationships were heterogeneous, possibly influenced by underlying factors beyond volume alone, including complexity and patient/disease characteristics.

### Non-Significant Associations

No demographic or perioperative variable, including age, comorbidities, stroke presentation, surgical side, or perioperative antiplatelet management, showed a statistically significant association with functional outcome, perfusion improvement, or bypass patency. While these findings did not meet formal thresholds for statistical significance, outcomes were clinically uniform, with no subgroup demonstrating disproportionately adverse results. This pattern likely reflects both sample size limitations and the stabilising effect of standardised surgical and perioperative care.

### Centralisation, Training, and National Implications

The centralised surgical model at our institution—characterised by a small number of high-volume surgeons, with all bypass procedures performed by the same two dedicated neurosurgeons and supported by standardised perioperative protocols—likely contributed to the consistency of outcomes and the low complication rates observed. Beyond clinical outcomes, this experience highlights the feasibility of developing a national referral framework for moyamoya disease in Malaysia, with structured training pathways and standardised decision-making. Formalising these practices into a departmental and potentially national protocol may help reduce variability in care, improve access to revascularisation, and support the development of future neurovascular specialists.

### Limitations

This study is limited by its retrospective design, single-centre setting, and relatively small sample size, constraining the ability to perform multivariable predictive modelling. The low incidence of adverse outcomes, while clinically reassuring, limited the statistical power to identify independent risk factors. Radiological follow-up was not uniform across all patients, especially in a small number of cases, reflecting real-world practice. The retrospective design limited accurate determination of the interval between symptom onset and surgical intervention. Although all patients were symptomatic, variability in symptom recognition, referral pathways, and medical record documentation precluded reliable calculation of the median time to surgery and assessment of its impact on outcomes. Nevertheless, the consistency of surgical technique, centralised case management, and comprehensive clinical follow-up strengthened the validity of our findings.

## Conclusion

The institutional experience at Hospital Kuala Lumpur stands as a compelling testament to how strategic training, operative consistency, and progressive standardisation can culminate in outstanding clinical outcomes. With a bypass patency rate nearing 95% and the majority of patients regaining functional independence, the department has laid a robust foundation for cerebral revascularisation in Malaysia. This dataset not only reinforces the viability of STA–MCA bypass for moyamoya disease, but also provides a compelling argument for the development of a formalised national protocol, with Hospital Kuala Lumpur as a leading reference centre.

Despite these limitations, our experience demonstrates that cerebral bypass surgery for moyamoya disease can be performed safely and effectively in a maturing neurosurgical programme. High rates of functional recovery, perfusion improvement, and graft durability support the role of STA–MCA bypass as a sustainable treatment strategy at a national referral centre. These findings provide a foundation for ongoing protocol refinement and contribute regional data to the growing global literature on adult moyamoya disease.

## Figures and Tables

**Figure 1:**
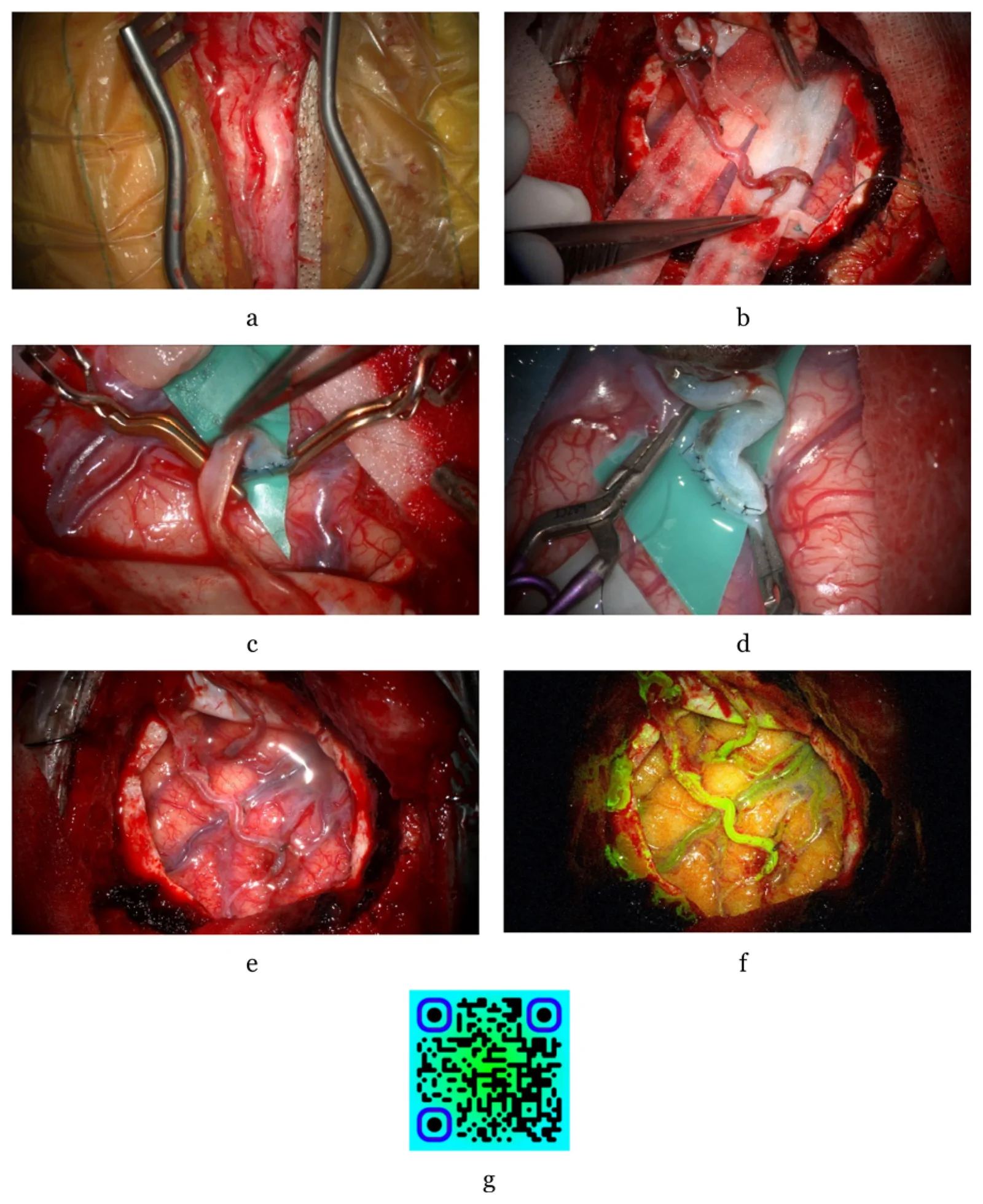
Technical steps for double-barrel STA-MCA bypass surgery: a) Vertical linear incision made and superficial temporal artery (STA) dissected; b) Preparation of donor (STA); c) End-to-side anastomosis of frontal branch of STA (donor) with M4 (recipient) at temporal lobe; d) Close view of example of end-to-side anastomosis of STA-MCA bypass; e) Double-barrel STA-MCA bypass; f) Double-barrel STA-MCA bypass under fluoresceine; g) Scan for our video of STA-MCA bypass surgery.

**Figure 2:**
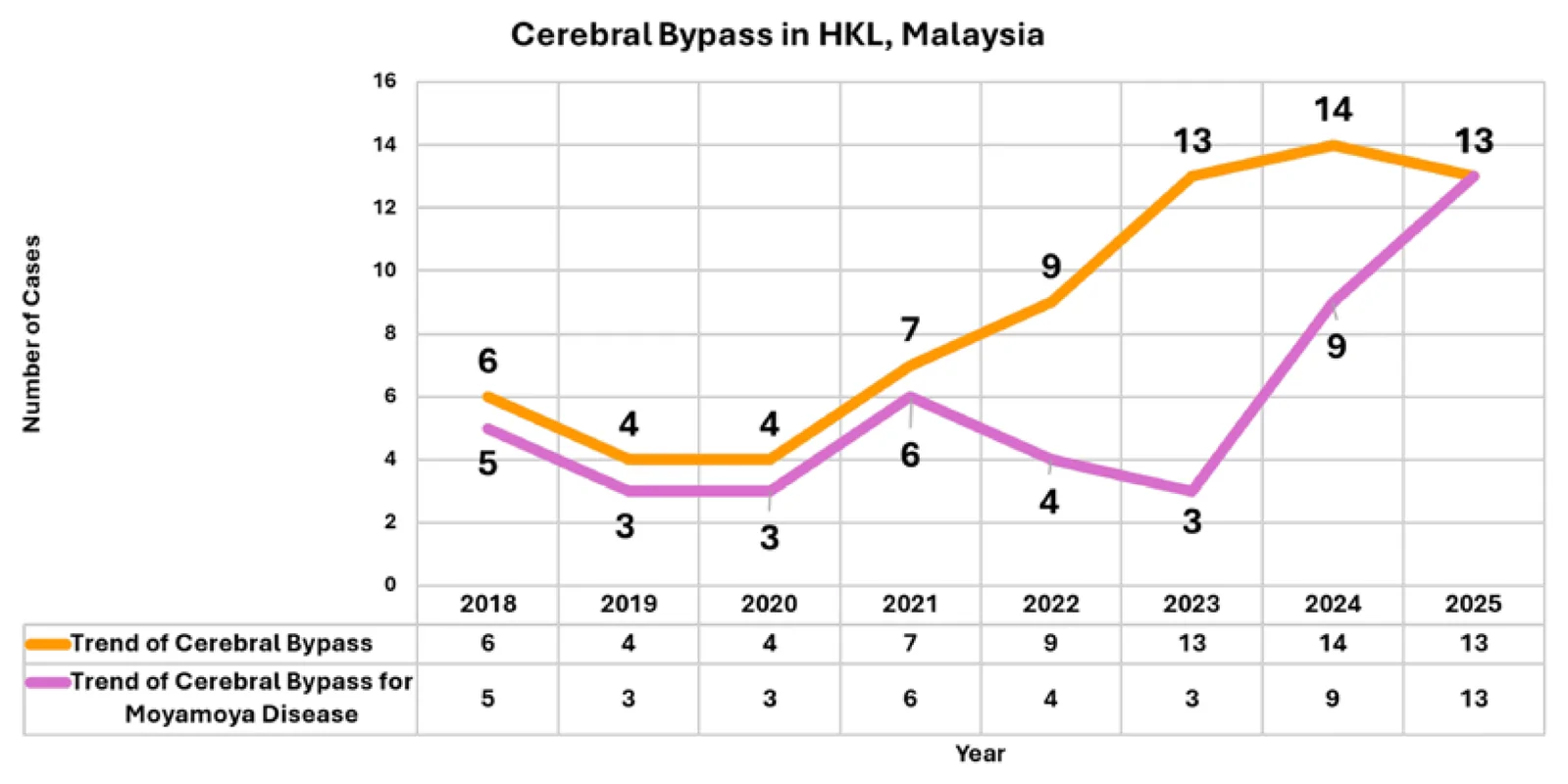
Graph showing cerebral bypass in Hospital Kuala Lumpur, Malaysia.

**Figure 3 f3-06mjms3304_oa:**
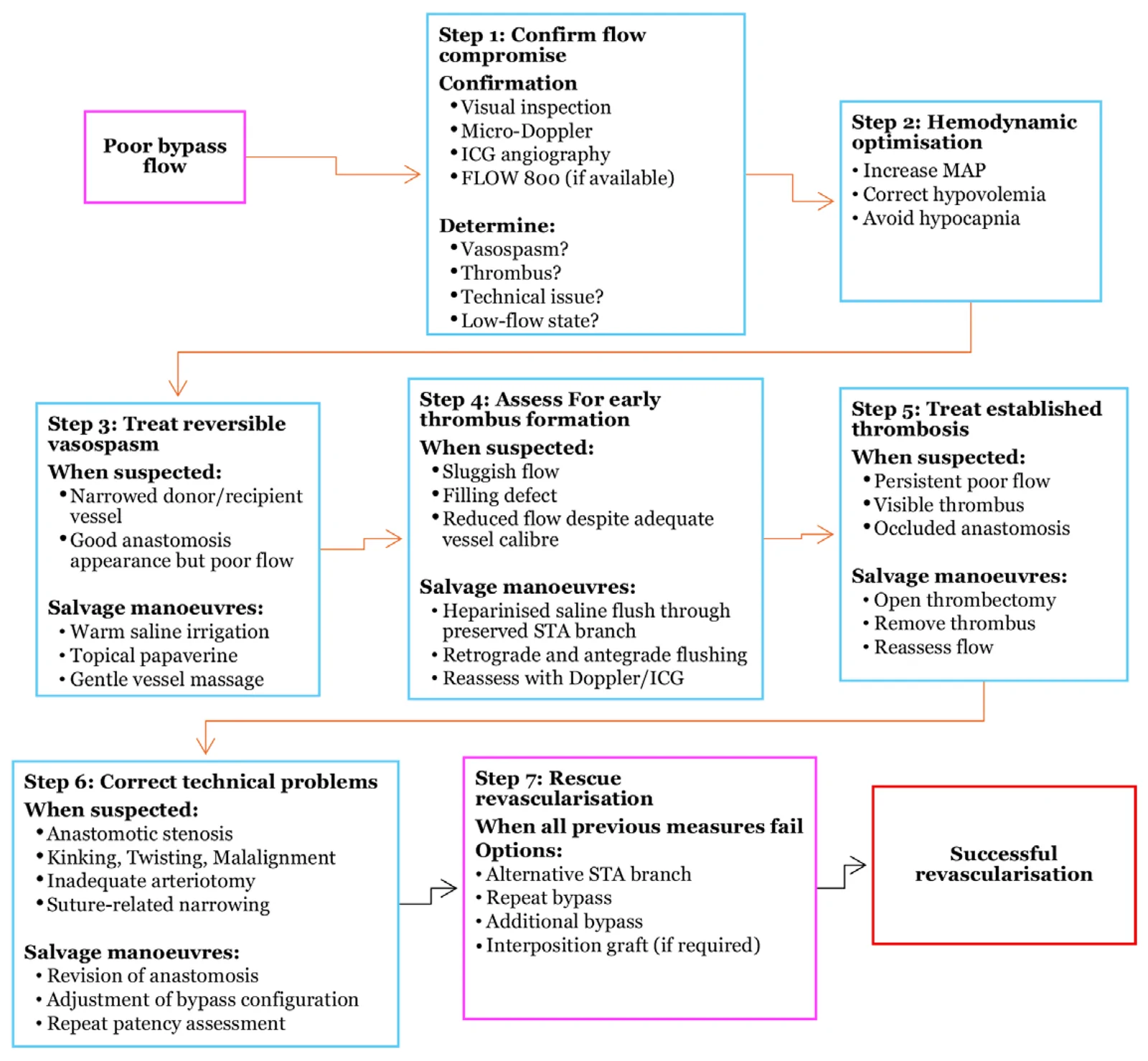
Intraoperative troubleshooting algorithm for STA–MCA bypass flow compromise.

**Table 1:** Overview of cerebral revascularisation surgeries in Hospital Kuala Lumpur, Malaysia.

Category	Details	Value	
Total neurovascular cases who receive interventions (2022–2025)	2022	176	
	2023	221	
	2024	175	
	2025	198	

Trend of bypass cases (2017–2025)	2017–2025	Breakdown as in Figure 2	
	Total	74	

Bypass aetiology, *n* = 61	Moyamoya	50	67.6%
	Aneurysm	16	21.6%
	Carotid stenosis	4	5.4%
	Others (tumour, stroke, etc.)	4	5.4%

Trend of bypass cases for moyamoya disease	2017–2025	Breakdown as in Figure 2	
	Total	50	67.6%

**Table 2:** Baseline characteristics of moyamoya patients undergoing STA–MCA bypass surgery (2017–2024), *N* = 37.

Variables				*n* (%)
**Demographic characteristics**				

Age (year old)	Age range	20 to 62		
	Median (IQR)	43.0 (32.0, 51.0)		

Age group (year old)	18 to 30			6 (16.00)
	30 to 60			30 (81.00)
	> 60			1 (3.00)

Gender distribution	Male			17 (46.00)
	Female			20 (54.00)

Ethnicity	Malay			18 (49.00)
	Indian			5 (13.00)
	Chinese			14 (38.00)
	Others			0 (0.00)

Comorbidities (general)	Yes			29 (78.00)
	No			8 (22.00)

Comorbidities (details)	Hypertension			22 (59.50)
	Dyslipidaemia			12 (32.40)
	Diabetes mellitus			9 (24.30)
	Ischaemic heart disease			1 (2.70)
	Others (CKD, NF, smoker)			7 (18.90)

Clinical presentation	Ischaemic stroke			28 (75.70)
	Haemorrhagic stroke			9 (24.30)

**Perioperative characteristic**				

Suzuki staging	Stage 1			0 (0.00)
	Stage 2			0 (0.00)
	Stage 3			5 (13.50)
	Stage 4			24 (64.90)
	Stage 5			8 (21.60)
	Stage 6			0 (0.00)

Perfusion study grading	Grade 1			0 (0.00)
	Grade 2			2 (5.40)
	Grade 3			33 (89.20)
	Grade 4			2 (5.40)

Surgery side	Right			16 (43.20)
	Left			21 (56.80)

Bypass type	Single			31 (84.00)
	Double			6 (16.00)

Surgeons	TKJ			35 (95.00)
	SAM			1 (2.70)
	MRMA			5 (13.50)
	RR			2 (5.00)
	AK			1 (2.70)
	LMS			1 (2.70)
	SAMZ			17 (46.00)
	MS			3 (8.00)

Antiplatelet withheld 5 to 10 days preoperatively	Yes			32 (86.50)
	No			5 (13.50)

Duration of surgery (hours)	Mean duration, hours (SD)	≈ 4.0 ± 0.6		
	Median duration, hours (IQR)	4.0 (4.0 to 4.0)		
	Minimum, hours	2		
	Maximum, hours	5		

Intraoperative patency	Immediate			29 (78.40)
	After adjunct manoeuvre^b^			8 (21.60)

**Postoperative outcome**				

**Clinical outcome**				

Postoperative clinical events		**< 3 months**	**> 3 months**	***n* (%)**
	
	Seizure	2	1	3 (8)
	TIA	3	0	3 (8)
	Minor Stroke	1	1	2 (5)
	Major Stroke	0	0	0 (0)
	Mortality	0	0	0 (0)

	Total	6	2	8 (21)

Three-month clinical outcome	Good (mRS 0 to 2)			35 (95.00)
	Poor (mRS 3 to 6)^a^			2 (5.00)

mRS distribution after three months	mRS 0			14 (38.00)
	mRS 1			17 (46.00)
	mRS 2			5 (14.00)
	mRS 3			1 (2.00)

**Radiological outcome**				

Perfusion study (CTP/MRP six months), *n* = 34, ano perfusion imaging done for three patients	Unchanged			4 (11.80)
	Improve			29 (85.30)
	Hyperperfusion			1 (2.90)

Bypass patency (DSA six months), *n* = 33, anot done for four patients	Patent			31 (94.00)
	Not patent			2 (6.00)

CKD = chronic kidney disease; NF = neurofibroma; SD = standard deviation; IQR = interquartile range; TIA = transient ischaemic attack; CTP = CT perfusion; MRP = MR perfusion; DSA = digital subtraction angiography.

a Presented with mRS 5, after 3 months improved to mRS 3;

b Adjunct manoeuvres included irrigation with warm saline or papaverine, vessel massaging or revision of the anastomosis, or additional technical adjustments as deemed necessary intraoperatively.

**Table 3:** Functional outcome over time assessed by modified Rankin Scale (mRS)

Time point	Good outcome (mRS 0 to 2), *n* (%)	Poor outcome (mRS 3 to 6), *n* (%)
Pre-operative	27 (73.0)	10 (27.0)
1-month postoperative	32 (86.5)	5 (13.5)
3-month postoperative	35 (95.0)	2 (5.0)

**Table 4:** Univariate associations between clinical variables and study outcomes.

Clinical variable	*P*-value (mRS outcomes)	*P*-value (radiological outcomes)	*P*-value (bypass patency)
Age (years)	0.788^b^	0.392^b^	0.500^b^
Gender	0.489^a^	0.652^a^	0.227^a^
Comorbidities	0.099^a^	0.389^a^	0.521^a^
Type of stroke presentation	0.054^a^	0.622^a^	0.616^a^
Surgical side	0.180^a^	0.397^a^	0.324^a^
Withheld antiplatelet therapy	0.745^a^	0.512^a^	0.716^a^
Duration of surgery (hours)	0.006^b^	0.857^b^	0.273^b^

a *P*-values calculated using Fisher’s exact test;

b *P*-values calculated using Mann–Whitney U test

**Table 5:** Association of preoperative antiplatelet holding and type of bypass with duration of surgery.

Clinical variables	*P*-value (preoperative antiplatelet withholding)	*P*-value (type of bypass – single vs. double-barrel)
Duration of surgery	0.011^b^	0.006^b^
Immediate intraoperative patency	0.564^a^	< 0.001^a^
Clinical event	0.567^a^	< 0.001^c^

a *P*-values calculated using Fisher’s exact test;

b *P*-values calculated using Mann–Whitney U test;

c *P*-values calculated using chi-squared

**Table 6:** Association of preoperative antiplatelet withholding with outcomes (with effect sizes).

Outcome	Withheld (%)	Not withheld (%)	Risk difference (RD)*	*P*-value
Intraoperative immediate patency	78.1	80.0	−1.9	0.564
Bypass patency at six months (DSA/CTA)	78.1	80.0	−1.9	0.564
Postoperative clinical event	6.3	0.0	6.3	1.000

* Risk difference = (withheld–not withheld); Positive values indicate higher rates in the withheld group.

**Table 7:** Surgeon experience (case volume) and outcomes.

Outcome	Result
Bypass patency at six months^a^	*P* = 0.480
Immediate intraoperative patency^a^	*P* = 0.480
Clinical event	*P* = 0.797
Duration of surgery (hours)^b^	ρ = −0.463, *P* = 0.0039
Outcome mRS (poor vs good)^a^	Not interpretable

a *P*-values calculated using Mann–Whitney U test;

b *P*-values calculated using Spearman correlation.

**Table 8:** Baseline disease severity markers according to bypass configuration.

Variable	Single-barrel, *n* = 31	Double-barrel, *n* = 6	*P*-value
CT perfusion grade			0.351^a^
Grade 2	1	1	
Grade 3	28	5	
Grade 4	2	0	
Suzuki stage			0.177^b^
Stage 3	3	2	
Stage 4 to 5	28	4	
Preoperative mRS			0.620^b^
Good, mRS 0 to 2	24	4	
Poor, mRS 3 to 6	7	2	

a Fischer-Freeman-Halton exact test;

b Fisher’s exact test;

c Fisher–Freeman–Halton exact test (extension of Fisher’s exact test for larger contingency tables) was performed due to the small sample size and low expected cell counts.
